# Multi-Target Directed Donepezil-Like Ligands for Alzheimer's Disease

**DOI:** 10.3389/fnins.2016.00205

**Published:** 2016-05-25

**Authors:** Mercedes Unzeta, Gerard Esteban, Irene Bolea, Wieslawa A. Fogel, Rona R. Ramsay, Moussa B. H. Youdim, Keith F. Tipton, José Marco-Contelles

**Affiliations:** ^1^Departament de Bioquímica i Biologia Molecular, Institut de Neurociències, Facultat de Medicina, Universitat Autònoma de BarcelonaBarcelona, Spain; ^2^School of Biochemistry and Immunology, Trinity Biomedical Sciences Institute, Trinity College DublinDublin, Ireland; ^3^Department of Hormone Biochemistry, Medical University of LodzLodz, Poland; ^4^Biomolecular Sciences, Biomedical Sciences Research Complex, University of St AndrewsSt. Andrews, UK; ^5^Department of Pharmacology, Ruth and Bruce Rappaport Faculty of Medicine, Eve Topf and National Parkinson Foundation Center for Neurodegenerative Diseases ResearchHaifa, Israel; ^6^Laboratory of Medicinal Chemistry, Institute of General Organic Chemistry, Spanish National Research CouncilMadrid, Spain

**Keywords:** multi-target-directed ligands, donepezil, oxidative stress, anti-β-amyloid aggregation, Alzheimer's disease

## Abstract

**HIGHLIGHTS**

- **ASS234** is a MTDL compound containing a moiety from Donepezil and the propargyl group from the PF 9601N, a potent and selective MAO B inhibitor. This compound is the most advanced anti-Alzheimer agent for preclinical studies identified in our laboratory.
- Derived from ASS234 both multipotent donepezil-indolyl (**MTDL-1**) and donepezil-pyridyl hybrids (**MTDL-2**) were designed and evaluated as inhibitors of AChE/BuChE and both MAO isoforms. **MTDL-2** showed more high affinity toward the four enzymes than **MTDL-1**.
- **MTDL-3** and **MTDL-4**, were designed containing the N-benzylpiperidinium moiety from Donepezil, a metal- chelating 8-hydroxyquinoline group and linked to a N-propargyl core and they were pharmacologically evaluated.
- The presence of the cyano group in **MTDL-3**, enhanced binding to AChE, BuChE and MAO A. It showed antioxidant behavior and it was able to strongly complex Cu(II), Zn(II) and Fe(III).
- **MTDL-4** showed higher affinity toward AChE, BuChE.
- **MTDL-3** exhibited good brain penetration capacity (ADMET) and less toxicity than Donepezil. Memory deficits in scopolamine-lesioned animals were restored by **MTDL-3**.
- **MTDL-3** particularly emerged as a ligand showing remarkable potential benefits for its use in AD therapy.

Alzheimer's disease (AD), the most common form of adult onset dementia, is an age-related neurodegenerative disorder characterized by progressive memory loss, decline in language skills, and other cognitive impairments. Although its etiology is not completely known, several factors including deficits of acetylcholine, β-amyloid deposits, τ-protein phosphorylation, oxidative stress, and neuroinflammation are considered to play significant roles in the pathophysiology of this disease. For a long time, AD patients have been treated with acetylcholinesterase inhibitors such as donepezil (Aricept®) but with limited therapeutic success. This might be due to the complex multifactorial nature of AD, a fact that has prompted the design of new Multi-Target-Directed Ligands (MTDL) based on the “one molecule, multiple targets” paradigm. Thus, in this context, different series of novel multifunctional molecules with antioxidant, anti-amyloid, anti-inflammatory, and metal-chelating properties able to interact with multiple enzymes of therapeutic interest in AD pathology including acetylcholinesterase, butyrylcholinesterase, and monoamine oxidases A and B have been designed and assessed biologically. This review describes the multiple targets, the design rationale and an in-house MTDL library, bearing the *N*-benzylpiperidine motif present in donepezil, linked to different heterocyclic ring systems (indole, pyridine, or 8-hydroxyquinoline) with special emphasis on compound **ASS234**, an *N*-propargylindole derivative. The description of the *in vitro* biological properties of the compounds and discussion of the corresponding structure-activity-relationships allows us to highlight new issues for the identification of more efficient MTDL for use in AD therapy.

## Introduction

Alzheimer's disease (AD) is one of the most common neurodegenerative diseases accounting for more than 80% of total dementia cases in elderly people. It is estimated that currently 47 million victims of AD exist worldwide and that number is expected to grow up to more than 130 million by 2050 as a result of life expectancy increase over the next decades (Thies and Bleiler, 2012, 2013). In 2015, the World Alzheimer Report estimated that the current annual societal and economic cost of dementia was US $818 billion worldwide and that amount is expected to rise up to 1 trillion by 2018. This study also reported that the cost associated with dementia has increased by 35% since 2010.

The clinical manifestations of AD are characterized by misfunctioning and gradual neuronal death resulting in a progressive memory deterioration and cognitive decline, related with the loss of cholinergic dysfunction. The anatomopathology of AD has been described by progressive loss of synaptic neurons triggering atrophy in the hippocampus and frontal and tempo-parietal cortex. Two distinctive hallmarks of AD include the presence of accumulated amyloid beta (Aβ) plaques around neurons (Glenner and Murphy, 1989) and hyperphosphorylated microtubules associated with tau protein in the form of intracellular neurofibrillary tangles (NFT) (Goedert et al., 2006).

The pathogenesis of this neurodegenerative disorder is not yet fully understood, but the scientific consensus is quite firm in describing it as a multifactorial disease caused by several elements. These include loss of cholinergic transmission, excessive protein misfolding and Aβ aggregation (Terry et al., 1964; Grundke-Iqbal et al., 1986), oxidative stress and free radical formation (Coyle and Puttfarcken, 1993), metal dyshomeostasis (Huang et al., 2004), excitotoxic, and neuroinflammatory processes (Coyle and Puttfarcken, 1993). Although a large number of genes has been associated with the AD late-onset condition, these appear to affect susceptibility or rate of progression rather than being directly causative (Bertram and Tanzi, 2008; Chouraki and Seshadri, 2014; Karch and Goate, 2015).

## Cholinergic hypothesis

Cholinergic neurotransmission modulates cognitive function and cortical plasticity (Arendt and Bigl, 1986) and also plays key roles in the control of cerebral blood flow (Biesold et al., 1989), cortical activity (Détári et al., 1999), and the sleep-wake cycle (Lee et al., 2005). In 1971, Deutsch postulated the involvement of the cholinergic system in learning and memory (Deutsch, 1971), which was later corroborated in studies with animal models and humans (Fibiger, 1991; Schliebs, 2005).

The first physiological evidence for the involvement of the cholinergic system in AD pathology was a reduction in pre-synaptic acetylcholine (ACh) and a reduced expression of choline acetyltransferase (ChAT), the enzyme responsible for ACh synthesis. In addition, decreases in the binding parameters of both muscarinic acetylcholine receptors (mAChR) and nicotinic acetylcholine receptors (nAChR) have been reported. These findings suggest a direct link between cholinergic neurotransmission and AD, and constitute the basis for the so-called “cholinergic hypothesis of AD” (Deutsch, 1971), which the main therapeutic approach used to date to address the cognitive loss associated with AD is based on.

### Cholinesterases

Cholinesterases (ChE) catalyse the hydrolysis of ACh into choline and acetic acid, an essential process in the cholinergic neurotransmission. There are two types of ChEs: acetylcholinesterase (AChE, EC 3.1.1.7) and butyrylcholinesterase (BuChE, EC 3.1.1.8). Both enzymes are type α/β hydrolases folded with an α-helix bound with β-sheet containing a catalytic domain (Ollis et al., 1992).

### Acetylcholinesterase

AChE is expressed in cholinergic neurons and neuromuscular junctions where its primary function is the rapid breakdown of the neurotransmitter ACh released during cholinergic neurotransmission. Although AChE and BuChE are structurally similar, resembling each other by more than 50%, both their significance and location are substantially different.

**Figure 1:**
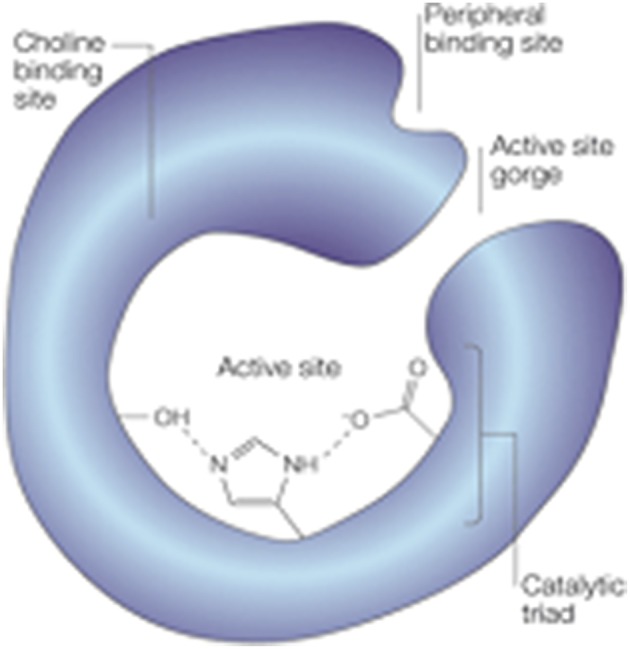
**Structure of AChE displaying the active site at the bottom of a narrow gorge, lined with hydrophobic amino-acid side chains within the catalytic triad, the choline binding site, and the peripheral binding site (Reproduced, with permission from Soreq and Seidman, 2001)**.

The structure of AChE has been widely studied since the 1990s. The active site of the enzyme lies on a bottom of a long and narrow cavity of 20 Å deep and contains a catalytic triad with amino acid residues Ser200, His440 and Glu327 that catalyses the hydrolysis of the ester bond. The active site also includes an anionic site or α-anionic site that interacts with the quaternary ammonium atom of ACh, ensuring its correct orientation. A peripheral anionic site (PAS) or β-anionic site, located on the enzyme surface around the cavity entrance (Figure 1) has been described to play an important role in AD. This site was first recognized in the 1960s as a target for AChE activity modulators (see Sussman et al., 1991) such as toxins and promising drugs. Furthermore, interaction of the Aβ peptide with the PAS contributes to the formation of amyloid plaques by accelerating the aggregation process. Propidium iodide is an example of an inhibitor binding to this site (Inestrosa et al., 1996).

One of the most consistent changes associated with AD has been the degeneration of neurons from the cholinergic nuclei in the basal forebrain region and their terminals in the hippocampus (Struble et al., 1982). The neuronal atrophy has been associated with a loss of cholinergic markers, occurring specifically in the nucleus basalis of Meynert (Vogels et al., 1990). The cholinergic system function, responsible for the storage and retrieval of items in memory, is therefore highly impaired in AD pathology, which has been corroborated by some neuroimaging data (Jack et al., 2010).

### Butyrylcholinesterase

BuChE is expressed in the hippocampus and temporal neocortex but at lower levels than AChE, and associated with glial cells (Mesulam et al., 2002), suggesting that these enzymes may have complementary functions. In AD brain, neuritic plaques, and NFTs contain large amounts of AChE and BuChE, the latter being present in the neuroglia (Wright et al., 1993), Moreover, these enzymes have been reported to play a role in the processing of APP (Wright et al., 1993), although it is unclear whether their inhibition could influence the pathogenic course of AD. While AChE is expressed in nerve and blood cells, hydrolysing ACh, the biological significance of BuChE still remains poorly understood, although it has been reported to partially modulate or compensate for the diminished AChE activity in deficient animals (Xie et al., 2000).

Likewise AChE, glial BuChE hydrolyses ACh into choline and acetate (Daikhin and Yudkoff, 2000; Mesulam et al., 2002) but with a different kinetic behavior. While AChE predominates in neurons and exhibits high affinity for ACh, with low *K*_*M*_ values, BuChE is mainly present in endothelia, glia and neuronal cells with low affinity (high *K*_*M*_ value) for ACh (Soreq and Seidman, 2001).

In addition to its role in the hydrolysis of ACh, non-enzymatic functions have also been attributed to BuChE. Whereas, AChE may accelerate amyloid deposition in the brain of AD patients, as previously mentioned, BuChE can associate with Aβ protein possibly delaying the onset and rate of neurotoxic Aβ fibril formation as observed *in vitro* (Diamant et al., 2006).

Activity of BuChE has been found either unaltered or increased in certain AD brain regions (Perry et al., 1978; Ciro et al., 2012). The increase has been associated with amyloid plaques and NFTs (Geula and Mesulam, 1989; Guillozet et al., 1997). In addition to changes in activity, changes in AChE and BuChE protein expression also occur during the progression of AD. An increase in the levels of glial-derived BuChE and decrease in synaptic AChE have been observed, triggering a dramatic increase in the BuChE: AChE ratio in cortical regions from 0.6, in healthy conditions, to 11 in AD pathology (Giacobini, 2003). The observed changes in BuChE activity and expression throughout the course of AD, and its relationship with cognitive function, emphasize the potential value of BuChE and AChE inhibition as therapeutic targets in AD condition.

## Amyloid hypothesis

The amyloid cascade hypothesis postulates that neurodegeneration in AD is caused by abnormal accumulation of Aβ plaques in various areas of the brain (Hardy and Higgins, 1992; Evin and Weidemann, 2002). This accumulation acts as a pathological trigger for a cascade that includes neuritic injury, formation of NFTs via tau protein to neuronal dysfunction and cell death (Hardy and Higgins, 1992; Selkoe, 1994). Genetic, biochemical, and pathological evidences support this hypothesis as the primary cause of AD (Kayed et al., 2003). The Aβ senile plaques are composed by Aβ peptides, which consist of 39–43 amino acid residues proteolytically derived from the sequential enzymatic action of β- and γ-secretases on transmembrane APP (Coulson et al., 2000). The length of Aβ peptides varies at the C-terminal according to the cleavage pattern of APP, with Aβ_1−40_ being the most prevalent form, followed by the hydrophobic form Aβ_1−42_ that aggregates faster (Perl, 2010). Within plaques, Aβ peptides and β-sheet conformation assemble and polymerise into structurally distinct forms such as fibrils, protofibers, and polymorphic oligomers (Selkoe, 1994).

The kinetics of the aggregation process of Aβ peptide follows a sigmoidal curve owing to the presence of β-sheets in its structure (LeVine, 1993), and it can be monitored *in vitro* by the use of dying molecules. The aggregating process comprises two main phases: lag phase and elongation phase. Over the lag or nucleation phase, soluble monomers or dimers with random-coil structures form a nucleus that proceeds rapidly to the formation of fibrils. At the end of this phase, low-weight soluble oligomeric species are formed, which are spherical, globular, and described as micelles or amorphous aggregates. The molecular mass of these species varies between 25 and 50 KDa (Lambert et al., 1998). Throughout the elongation phase, the oligomeric species link together to form high-molecular weight oligomers of up to 1000 KDa approximately, (Huang et al., 2000). These prefibrillar species transform into protofilaments or protofibrils, which are short, flexible, and fibrillar. The protofibrils are the precursors of full-length fibrils that are formed by simple lateral association and structural organization. Mature fibrils are straight, unbranched, twisted and can reach up to 10 μm in length (Dobson and Batich, 2004).

The Aβ aggregation process is highly susceptible to many factors, including pH, ionic strength of the solvent, purification process or temperature. These are responsible for complications in reproducibility when experimentally assayed *in vitro*. Distinct oligomerisation and assembly processes between Aβ_1−40_ and Aβ_1−42_ have been described (Bitan et al., 2003). While Aβ_1−42_ exhibits higher neurotoxicity and is able to form hexamers, heptamers, or octamers, Aβ_1−40_ reaches equilibrium from monomers to tetramers. These variances are related to differences in the Ile-41-Ala-42 dipeptide at the C-terminus of Aβ. Until recently, it was assumed that fibrillary Aβ deposits were the responsible agents for the neuronal injury and neurodegenerative process of AD (Hardy and Higgins, 1992; Selkoe, 1994). However, later findings showed that soluble oligomeric species were able to disrupt synaptic function (Lambert et al., 1998) and recent data support the belief that soluble dimeric species are highly toxic (Jin et al., 2011). The mechanism by which Aβ induces cell damage has been reported to be caused by reactive oxygen species (ROS) production (Schubert et al., 1995), altered signaling pathways (Mattson, 1997), mitochondrial dysfunction (Shoffner, 1997), and interaction with biometals (Jin et al., 2011).

**Figure 2:**
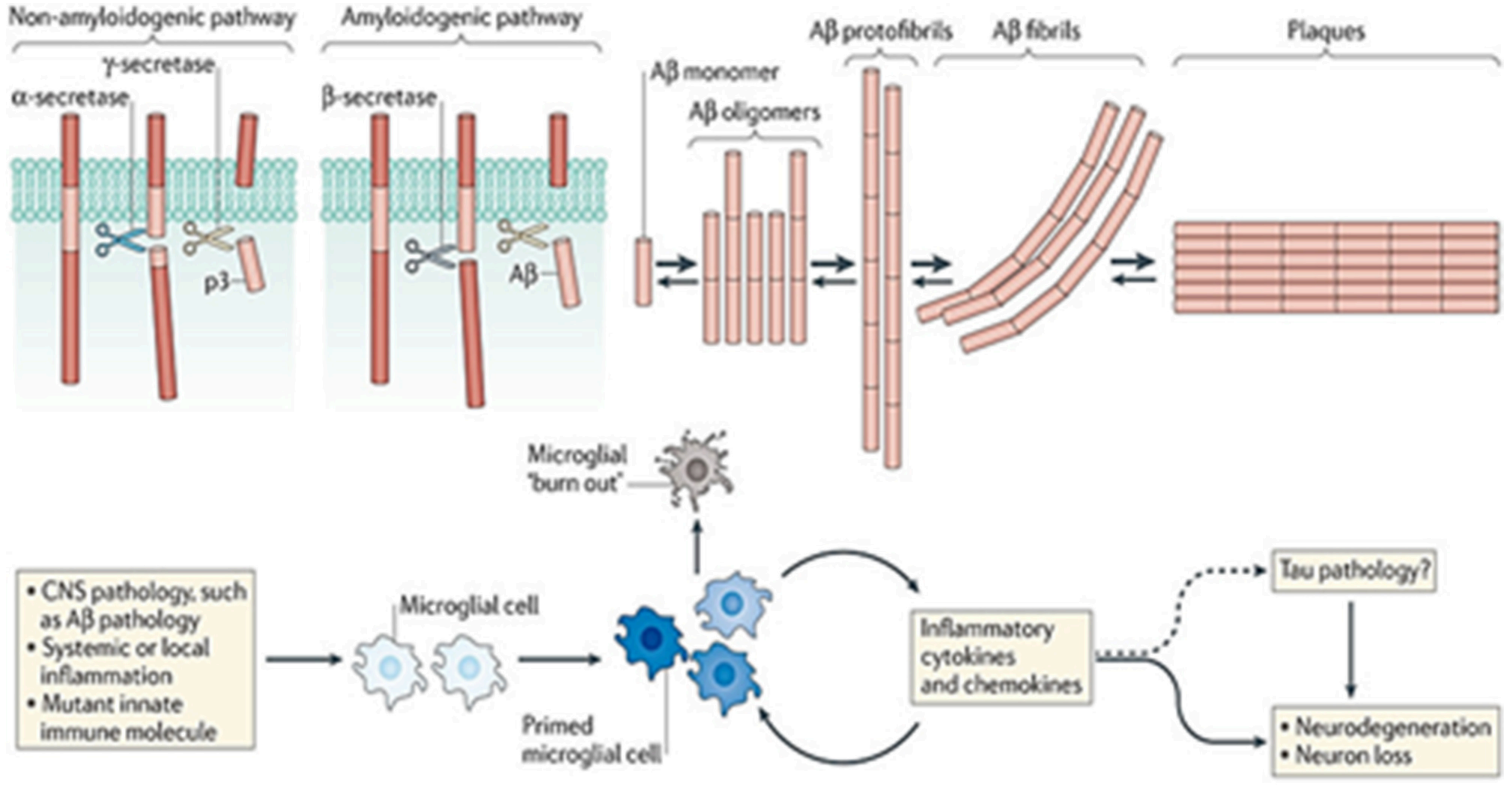
**Role of APP cleavage processing in AD and microglial activation (Reproduced, with permission from Heppner et al., 2015)**.

Both Aβ deposition and plaque formation also lead to local microglia activation, cytokine release, reactive astrocytosis, and multi-protein inflammatory responses (Heppner et al., 2015; Figure 2). The multifaceted biochemical and structural changes in surrounding axons, dendrites, and neuronal cell bodies also induce synapse loss as well as a remarkable cerebral atrophy in AD (Braak and Braak, 1994).

Nonetheless, to date no correlation between Aβ accumulation with extent of neuronal loss and cognitive dysfunction has been reported. In addition, direct Aβ-peptide neurotoxicity has been difficult to be identified in animal models, suggesting the existence of key intermediates between amyloidosis and neurodegeneration (Nelson et al., 2012; Serrano-Pozo et al., 2013). In addition to these studies, genetic research has suggested that neurodegenerative processes occurring in AD are the consequence of an imbalance between Aβ peptide production and clearance. Since the postulation of the amyloid hypothesis of AD, extensive but, so far unsuccessful, efforts have been undertaken in clinical research in order to develop novel therapeutics, this hypothesis has continued to gain support over the last two decades, particularly from genetic studies.

## Oxidative stress

Within any functional aerobic cell, the process involved in respiration inevitably generates ROS (Petersen et al., 2007). In particular, redox reactions are necessary for the generation of ATP and free radical intermediates are produced via the establishment of a proton gradient in oxidative phosphorylation. Multiple damaging mechanisms coexist in AD pathology, affecting each other at multiple levels (Von Bernhardi and Eugenín, 2012). In this respect, oxidative stress, which could be secondary to several other pathophysiological events, appears to be central in the progression of AD pathogenesis. Experimental evidence indicates that disturbances of the tissue redox state are important factors in early-stage AD, including the activation of multiple cell signaling pathways that contribute to the initial progression of the neurodegenerative process (Feng and Wang, 2012).

**Figure 3:**
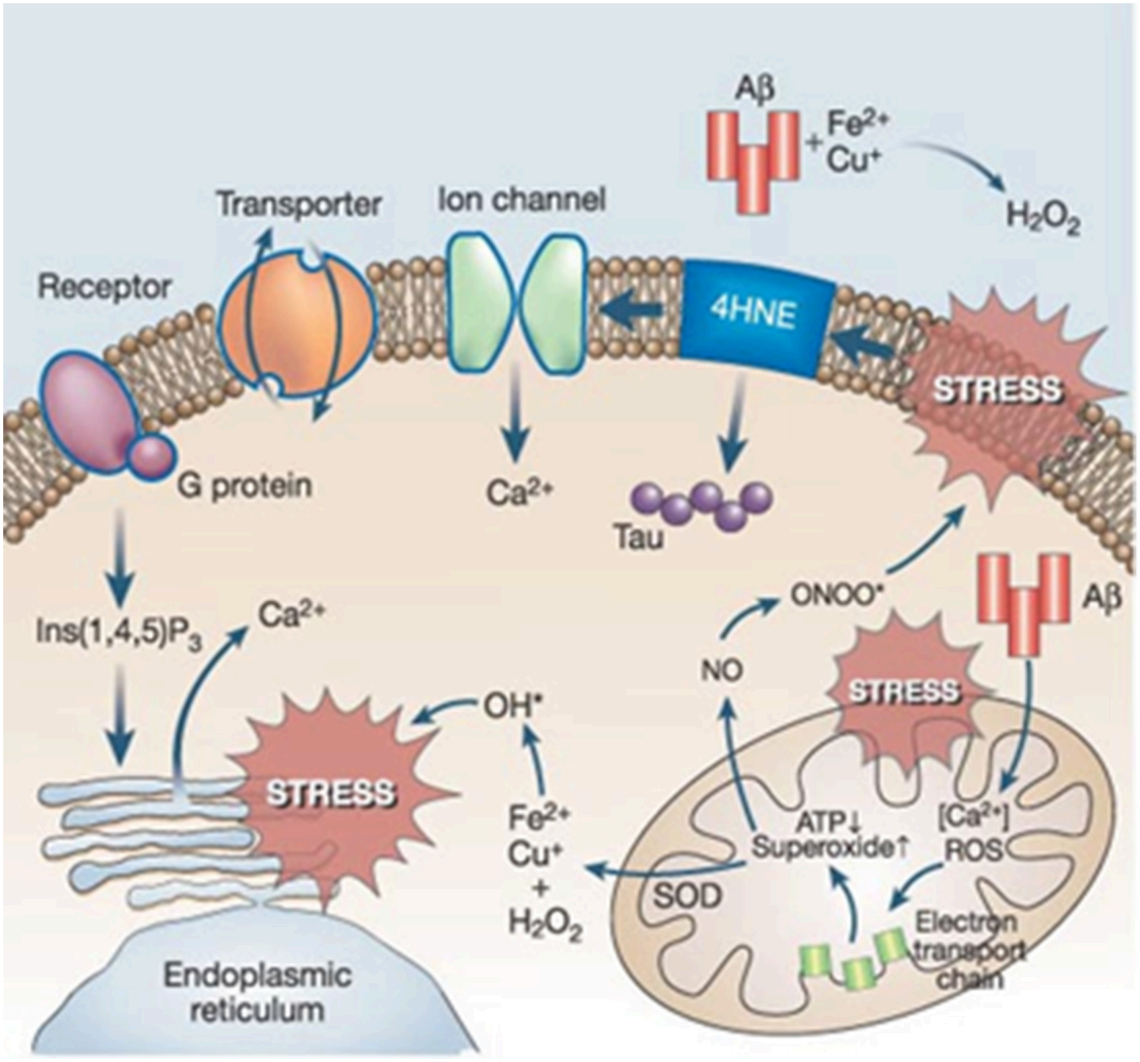
**Prevailing connections between oxidative stress and other key players in AD (Reproduced, with permission from Mattson, 2004)**.

Evidence of ROS and reactive nitrogen species (RNS)-mediated injury has been reported in AD (Praticò, 2008). Increased levels of oxidative damage to biomolecules including proteins, lipids, carbohydrates, and nucleic acids have been detected in a number of studies (Moreira et al., 2008; Fukuda et al., 2009; Sultana et al., 2011). In addition, levels of antioxidant enzymes were found to be altered in specific AD brain regions (Sultana et al., 2011). Consequently, the “oxidative stress hypothesis of AD” emerged as a key factor in both the onset and progression of the disease. Oxidative stress is a widespread cellular process that currently lacks a specific treatment target such as a receptor or a single major metabolic pathway (Galasko et al., 2012). The wide variety of sources and sites of oxidative stress production paralleled by an even higher heterogeneity in the antioxidant responses. Specifically, the activity of cytochrome oxidase and pyruvate and α-ketoglutarate dehydrogenase complexes (key enzymes for the ATP supply) have been reported to be decreased as a result of oxidative damage (Aliev et al., 2011). In AD, there are established connexions between oxidative stress and other key AD events that amplify its complexity (Figure 3).

The presence of oxidation markers in the early stages of AD indicates that oxidative stress precedes the appearance of some other hallmarks (Rosini et al., 2014). Moreover, the accumulation of oxidative active modified products, such as 8-hydroxyguanosine (8-OHG) and nitrotyrosine in the cytoplasm of cerebral neurons from Down's syndrome patients has been reported to precede amyloid deposition (Odetti et al., 1998; Nunomura et al., 2000). This finding was also demonstrated in an APP transgenic mice model of AD (Smith et al., 1998). In post-mortem brains of myocardial infarction (MCI) patients, CSF, plasma, and urine increased levels of lipid peroxidation, nuclear acid oxidation, and diminished levels of antioxidant enzymes were found (Keller et al., 2005; Butterfield et al., 2006). Furthermore, heme oxygenase-1 (HO-1) levels were also reported to be increased in both AD and MCI post-mortem brain tissues (Schipper et al., 2006). Such findings might indicate increased oxidative stress as a general response to tissue damage in the brain, which may therefore contribute to its further progression.

Although Aβ and its aggregated senile plaques are undoubtedly involved in the neurodegenerative process of AD, the chronology of their presence has been now deemed secondary by many studies (Castellani et al., 2006; Zhu et al., 2007). Evidence suggests that secretion and deposition of Aβ within the neurons are compensatory measures taken by cells in effort to protect themselves against damage triggered by oxidative stress (Hayashi et al., 2007; Nakamura et al., 2007). Mitochondrial abnormalities, initially caused by gradual oxidative disturbances are major contributors of ROS to the cell (Bonda et al., 2010). Oxidative perturbances in mitochondrial operations result in impaired metabolic capacity (Wang et al., 2009) that would prevent the effective transfer of electrons along the respiratory chain during oxidative phosphorylation and the further generation of excessive ROS. This abnormality creates a vicious positive-feedback cycle where ROS produce oxidative stress that eventually yields more ROS along with other cellular detriments. ROS gradually accumulate in the cell and once a threshold is reached, it is no longer able to control the debilitating cycle propagation and a compensatory “steady state” is unavoidably initiated in order to regain control of its environment (Zhu et al., 2007). This hyper-defensive state, intended to prolong cell life, increases vulnerability to additional insults such as Aβ peptides. The cell therefore becomes subject to oxidative damage produced by oligomerisation and aggregation (Wang et al., 2009), that in turn, leads to neuroinflammation, mitochondrial damage, and further ROS generation.

In addition, cellular oxidative damage has also been linked to tau hyperphosphorylation and formation of NFTs (Lee et al., 2004). As a consequence of this aberrant cycle, cells succumb to neurodegeneration exhibiting the distinctive cognitive decline and dementia descriptive of AD (Zhu et al., 2007). Altogether, the primary role of oxidative stress in both AD onset and progression open the possibility of developing specific disease-modifying antioxidant approaches to confronting the disease.

## Biometal dyshomeostasis

Numerous studies in AD and other neurodegenerative disorders have described an increase in the levels of oxidative stress reflected by a deregulated content of metals iron, copper, and zinc in the brain of patients. Recent findings have strongly pointed to brain oxidative stress as one of the earliest changes in AD pathogenesis that might play a central role in the disease progression (Guglielmotto et al., 2010; Lee et al., 2010). Redox-active metals, especially Fe^2+^ and Cu^2+^ are capable of stimulating free radical formation via the Fenton reaction, thereby increasing protein and DNA oxidation and enhancing lipid peroxidation (Khalil et al., 2011).

Metal ions have also been reported to mediate Aβ toxicity in AD (Duce et al., 2010). The Aβ peptide itself has been shown to be a strong redox-active catalyst able to produce hydrogen peroxide and OH^−^ in presence of copper or iron, which, in turn, are enriched in the amyloid cores of senile plaques (Huang et al., 1999, 2004). Metal ions can also interact with Aβ peptide enhancing its self-aggregation and oligomerisation at low physiological concentrations or under mildly acidic conditions (Huang et al., 1999). Moreover, metals can promote tau hyperphosphorylation and subsequent formation of NFTs by inducing aggregation upon tau interaction with Aβ (Yamamoto et al., 2002).

The presence of an iron-responsive element (IRE) in the 5′ untranslated regions (UTR) of AβPP mRNA was revealed as another link between iron metabolism and AD (Rogers et al., 2002; Silvestri and Camaschella, 2008). Iron closely upregulates intracellular levels of APP holo-protein by a mechanism that is similar to the translational control of ferritin L- and H- mRNAs through an IRE in their 5′ UTR (Rogers et al., 2002; Silvestri and Camaschella, 2008).

### The role of copper in AD

Copper is an essential trace element for all living animals. Copper levels are controlled by homeostatic mechanisms to prevent an excess, or a deficiency, that may trigger a unique set of adverse health effects. Copper can undergo redox cycling between Cu^1+^ and Cu^2+^ and the activities of some copper-containing enzymes, including superoxide dismutase (SOD), cytochrome-c oxidase, ceruloplasmin, and tyrosinase, have important biological functions. However, copper is also involved in the formation of free radicals by the Fenton reaction, in which free copper catalyses the formation of toxic hydroxyl radicals from physiologically available hydrogen peroxide (Barnham et al., 2004). Two classic disorders related to copper metabolism are Menkes and Wilson diseases, in which neurodegeneration is a common complication (Kodama et al., 1999; Merle et al., 2007).

In AD pathology, copper is mislocalized in brains, where decreased levels of this metal have been reported in affected regions (Deibel et al., 1996; Magaki et al., 2007) with enrichment in amyloid plaques and tangles (Lovell et al., 1998). Copper is released into the glutamatergic synaptic cleft facilitated by the copper-transporting P-type ATPase ATP7A at concentrations of ~15 μM (Hartter and Barnea, 1988; Hopt et al., 2003). There, it causes S-nitrosylation of NMDA receptors, inhibiting their activation (Schlief et al., 2005, 2006).

Aβ toxicity has been linked to the presence of redox metals, especially the binding to copper as well as the non-redox zinc ions (Hou and Zagorski, 2006; Tougu et al., 2008), although the precise mechanisms involved are still under investigation (Syme et al., 2004; Ma et al., 2006; Minicozzi et al., 2008; Alies et al., 2011). The copper binding site to Aβ_1−42_ has high affinity (*K*_*d*_ = 7.03 10^−18^ M; log*K*_*app*_ = 17.2), as measured by competitive metal-capture analysis, whereas that of Aβ_1−40_ is 10.3 (Atwood et al., 2000). The binding to copper has also been shown to modify and accelerate Aβ aggregation.

Copper also promotes dityrosine cross-linking of Aβ, which further accelerates Aβ aggregation (Atwood et al., 2000; Ali et al., 2005). The copper-induced Aβ oligomer contains a membrane-penetrating structure with histidine bridging (Curtain et al., 2001; Smith et al., 2006). This fact underlines the importance of histidine in the copper-Aβ interaction, which results in oligomers rather than the fibrils that are formed in the presence of zinc (Jiao and Yang, 2007). The copper-Aβ complex has been reported to exhibit cytotoxic properties (You et al., 2012) as this binding increases Aβ-induced cell death in cell culture (Wu et al., 2008; Perrone et al., 2010). Some hypotheses for this finding include the inhibition of cytochrome c (Crouch et al., 2005) and the increase of oxidative stress, triggered by the generation of hydrogen peroxide by Aβ and copper via a catalytic cycle (Huang et al., 1999). APP also interacts with copper by binding it between residues 142 and 166 (White et al., 1999; Barnham et al., 2003; Cappai et al., 2004) and catalytically oxidizing Cu^1+^ to Cu^2+^ (Multhaup et al., 1996). Furthermore, copper promotes APP internalization, whereas copper deficiency promotes Aβ secretion but not APP cleavage (Acevedo et al., 2011). Therefore, APP may not directly influence copper homeostasis, but inappropriate interaction with copper may be neurotoxic.

Tau phosphorylation and aggregation may also be induced by copper. Certain fragments in the four-repeat microtubule-binding domain of tau were shown to aggregate in the presence of copper *in vitro* (Ma et al., 2006; Zhou et al., 2007). Copper binding to tau induces hydrogen peroxide production *in vitro* (Su et al., 2007) and NFTs have also been shown to bind to copper in a redox-dependent manner as a source of ROS within the neuron (Sayre et al., 2000). In addition, copper exposure has been reported to induce tau hyperphosphorylation promoting tau pathology in a mouse model of AD (Kitazawa et al., 2009).

### The role of zinc in AD

The brain is the main pool of zinc within the body (Frederickson, 1989). Zinc is an essential component for many enzymes and transcription factors. In healthy conditions, most of the zinc content is located in membrane-bound metalloproteins (MP I, II, and III), loosely bound to zinc within the cytoplasm, and vesicular zinc, enriched in synapses. It has several functional roles in signal transmission. Synaptic transmission releases high concentrations of zinc into the synaptic cleft (Frederickson, 1989; Vogt et al., 2000; Qian and Noebels, 2005), where it acts as an antagonist of GABA (Molnar and Nadler, 2001; Ruiz et al., 2004) and NMDA receptors (Paoletti et al., 1997; Traynelis et al., 1998; Vogt et al., 2000) and activates the G-protein-coupled receptor GPR39 (Besser et al., 2009).

Inconsistent reports of zinc levels in AD have been published. Early surveys of brain tissue found no difference in zinc levels between AD and controls. Yet, later studies showed a decrease in zinc levels in neo-cortex (Danscher et al., 1997), superior frontal and parietal gyri, medial temporal gyrus and thalamus and hippocampus (Corrigan et al., 1993; Panayi et al., 2002). However, in other reports elevated zinc levels in AD-affected amygdala, hippocampus and cerebellum, (Deibel et al., 1996; Danscher et al., 1997), olfactory areas (Samudralwar et al., 1995), and superior temporal gyrus (Religa et al., 2006) were described.

The cause of this apparent zinc dysregulation remains unknown, yet it may be involved in alterations of proteins such as metallothionein III, which is found in neurons and reduced in AD brains (Uchida et al., 1991; Yu et al., 2001). In contrast, metallothionein I/II was found increased in astrocytes of post-mortem and preclinical AD brains (Adlard et al., 1998). In AD pathogenesis, enriched zinc levels have been found in amyloid plaques (Bush et al., 1994c). Studies using microparticle-induced X-ray emission tomography demonstrated a three-fold in zinc concentrations-surrounding neuropils in the amigdala (Lovell et al., 1998). These findings were later corroborated by histochemistry (Suh et al., 2000), autometallographic tracing (Stoltenberg et al., 2005) and synchrotron-based infrared and X-ray imaging (Miller et al., 2006).

Aβ binds to zinc at residues 6–28 (Bush et al., 1993, 1994a,b,c), with up to three zinc ions bound to histidines 6, 13, and 14 (Damante et al., 2009), inducing the aggregation of Aβ into soluble precipitates (Bush et al., 1994c). Therefore, zinc, like copper, accelerates Aβ-induced toxicity and zinc sequestration into amyloid deposits (Bush et al., 1994c) (Faller et al., 2013), inducing loss of functional zinc in the synapses. This loss may contribute to cognitive decline in AD due to ZnT-3 depletion (Adlard et al., 2010).

Zinc may also interfere with APP processing and function as these processes are coordinated by secretases under the zinc regulation. This metal increases the synthesis of PS-1 (Park et al., 2001); however, the γ-secretase activity is inhibited by Zn^2+^ (Hoke et al., 2007). Zinc binds directly to Aβ, hiding its proteolytic cleavage site (Bush et al., 1994b) and therefore inhibiting its degradation by matrix metalloproteases (Crouch et al., 2009). Secreted Aβ is normally degraded by proteases such as neprilysin within a short period of time. However, zinc and other metals enhance the oligomerisation and accumulation of the amyloid protein.

After being incorporated into the membrane, the conformation of Aβ changes and it aggregates on the membranes. These aggregates are able to form channels, which unlike endogenous Ca^2+^ channels, are not regulated by standard channel blockers. Thus, a continuous flow of Ca^2+^ is initiated and disruption of calcium homeostasis triggers several apoptotic pathways, including free radical formation and tau phosphorylation leading to cell death. Conversely, zinc secreted into synaptic clefts, inhibits Aβ-induced Ca^2+^ entry, and thus confers a protective function in AD (Kawahara et al., 2014).

In agreement with these findings, *in vivo* studies revealed that high intake of dietary zinc triggered elevated expression of APP and enhanced amyloidogenic APP cleavage and Aβ deposition in APP/PS1 transgenic mice (Borchardt et al., 2000; Cuajuangco and Faget, 2003; Wang et al., 2010). Finally, zinc may also be involved in tau pathology as it is enriched in tangle-containing neurons (Suh et al., 2000). Zinc moderates translation of tau and modulates its phosphorylation by affecting the activities of GSK-3β, protein kinase B, ERK1/2, and c-Jun N-terminal kinase (An et al., 2005; Pei et al., 2006; Lei et al., 2011). Zinc can also bind to tau monomers, altering their conformation (Boom et al., 2009) and inducing both aggregation and fibrillation of this protein (Mo et al., 2009).

### The role of iron in AD

Iron is a fundamental element in biology for oxygen transport and energy metabolism. It is a transition element that exists in oxidative states from −2 to +8, although in biological systems only ferrous (Fe^2+^) and ferric (Fe^3+^) states normally exist. The cycling between ferric to ferrous states is used in biology for various redox reactions essential to life. However, deleterious reactions with oxygen, such as Fenton reaction, which is catalyzed by the free ferrous iron and may involve bound Fe(IV) (Freinbichler et al., 2009) are a source of oxidative stress (Zigler et al., 2015).

In the brain, iron is involved in development (Beard, 2003), metabolic and neurotransmitter systems (Agarwal, 2001). It is therefore regulated by multiple proteins, reflecting its involvement in different cellular functions. In AD brains, iron is enriched in both NFTs (Smith et al., 1998) and senile plaques (Goodman, 1953) with an estimated concentration of three-fold higher that of the normal neuropil levels (Lovell et al., 1998). Iron accumulation occurs in the cortex but not in the cerebellum (Andrasi et al., 1995; Duce et al., 2010). The iron storage protein ferritin binds to most iron within the brain and its levels increase with age and in AD (Bartzokis and Tishler, 2000). Transferrin (Tf) is an extracellular iron-transporting protein expressed in the brain that exchanges iron between cells. The complex Fe-Tf is endocytosed (Eckenroth et al., 2011) and iron is reduced to its ferrous state by an unknown ferrirreductase.

Recently, AD-associated APP was identified as a neuronal ferroxidase (Duce et al., 2010). APP-knockout mice exhibit iron accumulation in the brain and peripheral tissues and loss of APP ferroxidase activity in AD brains is coincident with iron retention in the tissues (Duce et al., 2010). Recent studies reported that iron-export capability of APP requires tau protein (Lei et al., 2012), which is involved in axonal transport (Lei et al., 2010) and binds to APP (Islam and Levy, 1997). Moreover, the loss of tau in mice is reported to cause age-related iron accumulation (Lei et al., 2012). The iron-regulatory system is disturbed in AD brains and ferritin has been reported to be elevated and co-localized with senile plaques expressed in astrocytes (Connor et al., 1992) and increased in frontal cortex of AD brains (Loeffler et al., 1995). However, the amount of APP was not significantly reduced in cortex (Duce et al., 2010) but both ferroxidase activity and soluble tau protein levels were observed to be decreased (Shin et al., 1992; Khatoon et al., 1994; Zhukareva et al., 2001; Van Eersel et al., 2009; Duce et al., 2010). Genetic factors have also been linked to higher susceptibility to iron burden in AD (Blázquez et al., 2007; Bertram and Tanzi, 2008; Percy et al., 2008).

Iron burden enriches around the senile plaque region (Meadowcroft et al., 2009) and promotes Aβ aggregation *in vitro* (Mantyh et al., 1993). Furthermore, iron-aggregated Aβ toxicity in cell culture (Schubert and Chevion, 1995) is mediated by ROS production (Rottkamp et al., 2001) or by the activation of Bcl-2/Bax-related apoptosis pathway (Kuperstein and Yavin, 2003). As mentioned above, iron also binds to tau protein, but only Fe^3+^ has reported to induce aggregation of hyperphosphorylated tau protein, which can be reversed by reducing Fe^3+^ to Fe^2+^ (Yamamoto et al., 2002) or by the use of iron-chelators (Amit et al., 2008). In AD brain, NFTs bind iron, which acts as a source of ROS within the neurons (Sayre et al., 2000). A decrease of tau phosphorylation in hippocampal neurons treated with Fe^3+^ (Egaña et al., 2003), corresponding with a decrease in CDK5 activity. Conversely, treatment with Fe^2+^ also resulted in hyperphosphorylation (Lovell et al., 2004) without inducing aggregation, possibly resulting from upstream activation of the ERK1/2, MAPK pathway (Muñoz et al., 2006; Huang et al., 2007). Recently, oxygen tension-dependent transcriptional factor, hypoxia inducible factor-1α (HIF-1α) has emerged as a potential target in neurodegenerative diseases. This protein regulates intracellular iron by binding to HIF-responsive elements (HREs) that are located within the genes of iron-related proteins such as Tf receptor and heme oxygenase-1 (Petousi and Robbins, 2014).

Recent research and clinical trials have confirmed that HIF-1α activation may be a potent strategy for postponing the pathogenesis and ameliorating the outcomes of AD. In this context, the use of iron chelators such as M30 has been reported to increase the levels of this protein by inducing the expression of its target genes VEGF and EPO. In addition, overexpression of HIF-1α has been shown to protect cultured cortical neurons from Aβ-induced neurotoxicity, through activating glycolytic and hexose monophosphate shunt-related enzymes. Therefore, an additional use of iron-chelators to overexpress HIF-1α may contribute to preventing neuron death and ameliorating the symptomatology of AD by inducing the expression of neuroprotection-related genes (Benkler et al., 2010).

Thus, in the context of AD pathology, the involvement of both oxidative stress and metal ions in AD (Ayton et al., 2013), indicates the use of antioxidant-chelators as potential beneficial agents in the prevention and treatment of the disease, by scavenging free radicals and, more importantly, by blocking their production, as well as restoring normal metal balance.

Although abnormal accumulation of metals has been reported in AD for many years, it has been only recently considered as a key early factor in AD, closely related to increased levels of both oxidative stress and amyloid beta-protein toxicity in Barone (2016). The development of metal-chelators has emerged, concurrently, as a novel pharmacological approach against this neurological disorder. In this regard Youdim and co-workers have reported a novel multifunctional molecule, M30, which combines iron-chelation with and monoamine oxidase (MAO) inhibitory properties as a possible drug for the treatment of AD (Zheng et al., 2005; Avramovich-Tirosh et al., 2007).

## Neuroinflammation

Inflammation is a defensive mechanism of the body against multiple threats such as infections and injury. It is a complex event that involves both soluble factors and specialized cells (Brown et al., 2007). Similar inflammatory processes occur in the brain and peripheral tissues. In the brain, glial cells, including astrocytes and microglia, undergo activation under pro-inflammatory conditions by the increase in the production of inflammatory cytokines in the CNS, which become deleterious and leads to progressive tissue damage in degenerative diseases (Morales et al., 2014). Infiltration by peripheral macrophages may also occur (Rogers et al., 1996).

In chronic disorders such as AD pathology, inflammation plays a critical role. It has been reported that insoluble fibrillar Aβ surrounding microglia, reactive astrocytes and dystrophic neurites contributes to the process of neuronal degeneration by initiating a series of cellular events which are able to elicit an immune response. Moreover, Aβ deposition in parenchyma and blood vessels has been described to trigger microglial migration and mediation of acute and chronic inflammatory response against the aggregates, thus inducing the production of nitric oxide (NO), ROS, pro-inflammatory cytokines such as tumor necrotic factor α (TNFα) or inteleukins-1β and -6 (IL-1β, IL-6) and prostaglandin E2 (PGE2), which eventually may promote neuronal death (Kitazawa et al., 2009).

## MAO as a target

One of the major observations in late AD is the loss of neuronal cells and brain shrinkage. Specific loss of dopaminergic cells has led to the use of deprenyl or selegiline as adjunctive therapy in PD to prevent removal of dopamine by metabolism (Birkmayer et al., 1977). The same principle applies in AD, where the decrease in all neurotransmitters is due mainly to a loss of neuronal connections.

Monoamine oxidases (MAO, EC 1.4.3.4) are flavin adenine dinucleotide (FAD)-containing enzymes that catalyse the oxidative deamination of primary, secondary, or tertiary amines, producing the corresponding aldehyde, hydrogen peroxide and ammonia. There are two isoforms of MAO present in mammals: MAO A and MAO B, which share ~70% sequence identity and are encoded by separate genes located on the X chromosome. The C-terminal regions of MAOs are transmembrane α-helices that anchor the enzymes to the mitochondrial outer membrane leaving the rest of the protein exposed to the cytoplasm. The entry of a substrate or inhibitor into the active site of MAOs is predicted to occur near the intersection of the enzyme with the surface of the membrane (De Colibus et al., 2005; Edmondson et al., 2007).

In selective MAO-knockout mice, the two MAO isoforms resulted in significantly different effects on both metabolism and behavior. Whereas, the lack of MAO A triggered aggressive phenotypes as a result of elevated levels of 5-HT and NA and lesser of DA (Chester et al., 2015), only the levels of 2-phenylethylamine were increased in MAO B-knockout mice, which exhibited traits such as sensation seeking, impulsiveness and extraversion. MAO A and MAO B were originally distinguished by their sensitivity to nanomolar concentrations of the acetylenic inhibitors clorgyline and *l*-deprenyl, respectively, as well as by their substrate specificities. The content of MAO varies over lifetime as MAO A appears before MAO B, the brain level of which increases dramatically after birth (Tsang et al., 1986; Strolin-Benedetti et al., 1992; Nicotra et al., 2004). MAO B levels continue to increase throughout the lifetime and in AD progression. In rat peripheral nervous system, MAO is localized in the endothelial cells of the endoneurial vessels, Schwann cells and unmyelinated axons of some neurons (Matsubayashi et al., 1986).

In human brain, MAO activity differs among regions. While the highest levels are found in basal ganglia and hippocampus, the lowest are observed in the cerebellum and neocortex (O'Carroll et al., 1983). The anatomical distribution of MAO isoforms in human brains were confirmed by positron-emission topography (PET) using intravenous ^11^C-labeled irreversible inhibitors (Fowler et al., 1987; Saura et al., 1992). Immunohistochemical studies have revealed that serotonergic neurons and astrocytes are rich in MAO B whereas catecholaminergic neurons mainly contain MAO A (Westlund et al., 1988). Caudate dopaminergic nerve endings contain only MAO A and small amounts of this isoform are also found in the serotonergic nerve terminals (O'Carroll et al., 1987). Some differences in the dopamine metabolism have been observed between species. For instance, only MAO A isoform is involved in rat brain, whereas MAO B is primary responsible for dopamine metabolism in human brain (Fornai et al., 1999).

Inhibitors of MAO, reversible and irreversible, equipotent on both isoforms or highly selective, have been used for more than 60 years for the treatment of depression (Mendelewicz and Youdim, 1983; Youdim et al., 2006). The most successful approach has been the use of selective irreversible inhibitors as exemplified by deprenyl, now used in PD therapy. The chemical moiety of this compound includes the propargyl group, which is readily incorporated into different scaffolds to cause MAO inhibition in compounds that are designed for other targets. This finding has been identified as one of the mechanisms of various MTDL to delay the progression of neurodegeneration as it will be discussed below.

## FDA-approved drugs for AD

**Figure 4:**
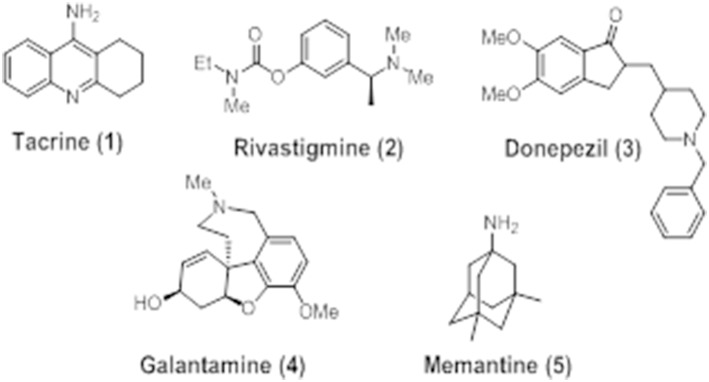
**Chemical structures of FDA-approved agents for use in AD: tacrine (1), donepezil (2), rivastigmine (3), galantamine (4), and memantine (5)**.

At present, only five drugs have been approved by the U.S. Food and Drug Administration (FDA, USA) for use in AD. These compounds are mainly based on the “cholinergic hypothesis” and aimed at re-establishing functional cholinergic neurotransmission. Four of these: tacrine (**1**), rivastigmine (**2**), donepezil (**3**), and galantamine (**4**) are cholinesterase inhibitors, whereas memantine (**5**) is a NMDA receptor antagonist (Figure 4).

### Tacrine

Tacrine or tetrahydroaminoacridine (THA), marketed as *Cognex*®, was the first drug to be approved for use in AD by the FDA in 1993. It is a competitive AChE inhibitor with high lipid solubility (Nielsen et al., 1989) also able to interact with muscarinic receptors (Adem, 1993) and MAO A and B (Adem et al., 1989). Although it had some relatively small benefits on cognition, tacrine was withdrawn from the market in 2013 due to the high incidence of side effects, mostly related to hepatotoxicity (Qizilbash et al., 1998).

### Rivastigmine

Rivastigmine, marketed under the trade name of *Exelon*® since 1998, is a non-selective pseudoreversible ChE inhibitor reported to produce enhanced benefits over AChE inhibition alone (Touchon et al., 2006; Bullock and Lane, 2007). A recent study using a transdermal patch reported a reduction in the prevalence of side effects as well as positive results after administering rivastigmine to mild-to-moderate AD patients. These studies reported better outcomes, in comparison with placebo group, for rate of cognitive function decline and daily living activities (Birks et al., 2015).

### Galantamine

Galantamine (*Razadyne*®) is a well-studied drug approved in 2001 for the treatment of AD, possessing a dual mechanism of action to attenuate the symptoms of cognitive decline in AD. It is a reversible selective competitive AChE inhibitor (Greenblatt et al., 1999) that is able to simultaneously modulate nAChERs (Dajas-Bailador et al., 2003). The main mechanism of action of this molecule is the ability to increase the content of ACh, enhancing cholinergic neurotransmission and therefore improving cognition in AD patients by delay of ACh catabolism at synapse. Moreover, galantamine has also demonstrated to directly interact with the nAChER as low-affinity agonist, allosterically binding to a distinct binding site from where nicotinic agonists, such as ACh, choline, or carbachol, bind (Akk and Steinbach, 2005). Moriguchi et al. (2003) showed that galantamine also potentiates the activity of NMDARs, so that the dual mechanism of action on both cholinergic and glutamatergic systems in AD patients may elucidate the improvement in cognition, memory, and learning that have been found in clinical trials with this drug (Wilkinson and Murray, 2001).

### Memantine

Memantine, marketed as *Namenda*® or *Ebixa*®, is a glutamatergic agent and the first and only NMDAR antagonist approved in 2003 for the treatment of moderate-to-severe AD and dementia. In clinical trials, memantine has shown significant benefits on cognition, function, and global status of AD patients. Moreover, it appears to be harmless and well-tolerated, with a safety profile compared to placebo (Doody et al., 2007). Memantine binds to NMDARs with a low-micromolar IC_50_ value. Furthermore, it exhibits poor selectivity among NMDARs subtypes, with under micromolar IC_50_ values for NR2A, NR2B, NR2C, and NR2D receptors expressed in *Xenopus* oocytes (Parsons et al., 1999). Although memantine was initially regarded as a poor drug candidate for AD therapy, the mechanisms by which exerts clinical benefits and its safe profile have attracted a great considerable interest in the field of medicinal chemistry (Lipton, 2006). In addition, memantine has also been reported to exhibit neuroprotective activities against Aβ toxicity (Tremblay et al., 2000; Miguel-Hidalgo et al., 2002; Hu et al., 2007), tau phosphorylation (Song et al., 2008), neuroinflammation (Willard et al., 2000), and oxidative stress (Figueiredo et al., 2013; Liu et al., 2013).

### Donepezil

Donepezil, marketed under the trade name of *Aricept*® in 1996, is a brain-permeable reversible non-competitive ChE inhibitor approved for use in AD (Birks and Harvey, 2006) and currently the most widely prescribed drug for the treatment of this disease. Donepezil is highly selective for AChE over BuChE activity (405:1) (Nochi et al., 1995). Compared to other approved AChE inhibitors, donepezil is similarly effective in ameliorating cognitive and functional decline in AD with comparable safety and tolerability. Moreover, beneficial effects have been observed at lower doses (5 mg/day), which is highly valuable at minimizing adverse reactions. Although clinical trials with donepezil have been reported, modest but reproducible, improvements in cognition and global functioning in treated patients, as compared to placebo. These effects were not permanent as patients, continued to exhibit a decline in cognitive functioning over time (Doody et al., 2007).

In recent years, the consistent failure of pharmacological approaches targeting traditional AD targets such as Aβ and tau protein together with the existing number of drugs to be tested in pre-dementia stages of the disease has left no effective disease-modifying treatment of AD in sight. Thus, additional benefits of donepezil might be revealed with the use of this molecule in combination with other active moieties.

## Multi-target-directed ligands (MTDL)

**Figure 5:**
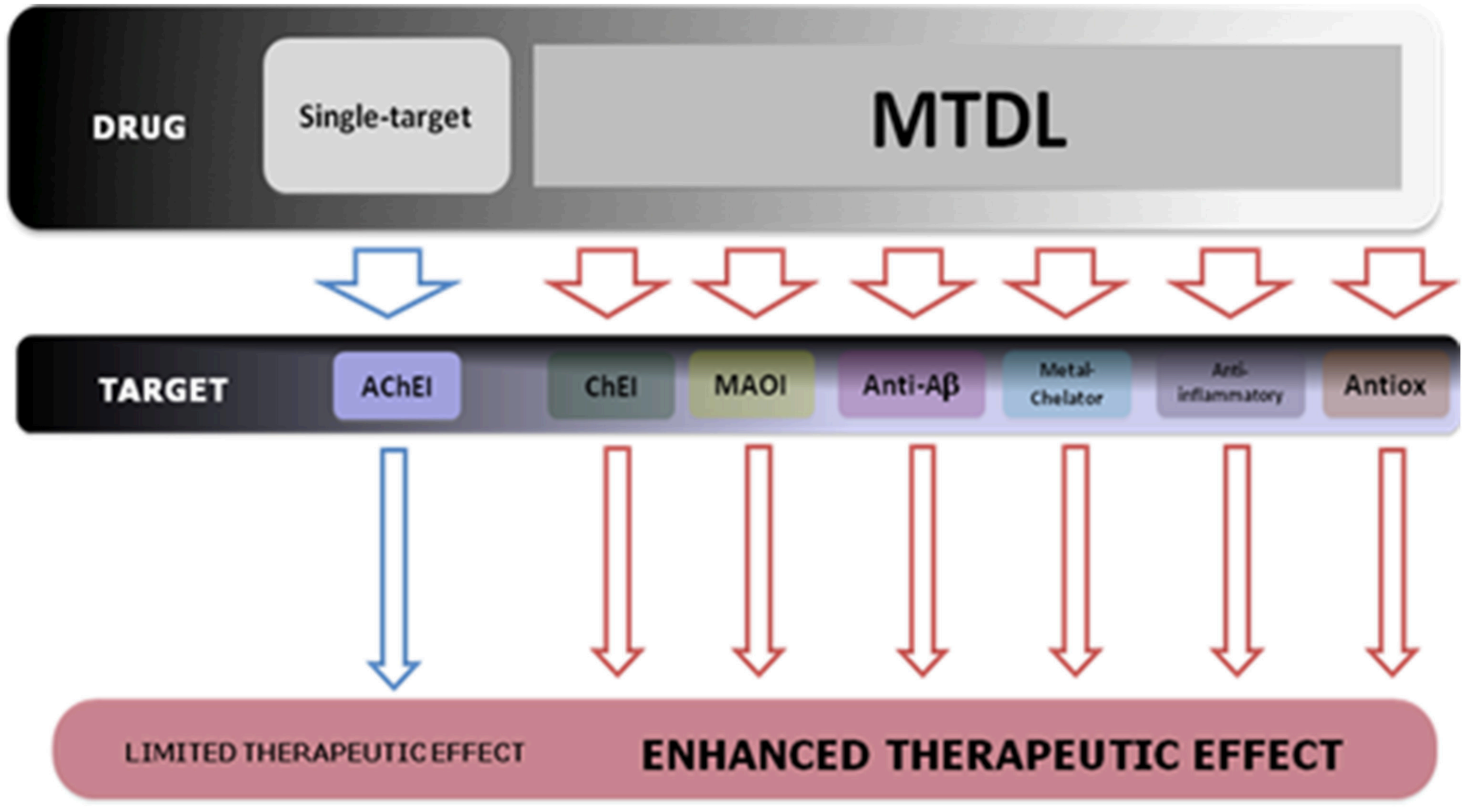
**Scheme of the therapeutic design strategy of MTDL for the treatment of the multifaceted nature of AD pathology**.

The lack of therapeutic effectiveness of the current FDA-approved drugs based on the single-target paradigm for the treatment of cognition and memory decline, prompted to the rational design and development of a novel and improved pharmacological approach against AD: the Multi-Target-Directed Ligands (MTDL) or “dirty drugs” (Buccafusco and Terry, 2000; Youdim et al., 2005; León et al., 2013). These molecules are conceived to directly interact weakly with multiple targets associated with AD by the molecular hybridisation of different pharmacophore moieties from identified bioactive molecules. The development of MTDLs obviates the challenge of simultaneously administering multiple drugs with potentially different degrees of bioavailability, pharmacokinetics, and metabolism. Furthermore, this pharmacological approach also provides AD patients with a simplification of the therapeutic regimen (Figure 5).

To date, the therapeutic potential of MTDLs for the treatment of complex neurodegenerative diseases and age-related cognitive impairment has not been realized. Multifaceted diseases such as AD, mainly caused by perturbations of the complex intracellular network that links to tissue and organs systems, needs a more comprehensive pharmacological approach. Therefore, approaches relying on targeting single proteins/mechanisms are not likely to have effective outcomes. Because of the existence of feedback mechanisms in biological systems, enzyme inhibition may not be able to decrease activity in long term, but instead, may result in increased activity, as reported from the use of rapid-reversible AChE inhibitors that significantly increased both protein activity and expression levels in the CSF of AD patients after long-term treatment (Darreh-Shori and Soininen, 2010).

Since 2005, literature has shown several promising results from applying this innovative approach to drug design. Well-known drugs such as donepezil, tacrine, or rivastigmine (Bolognesi et al., 2007; Samadi et al., 2011) as well as bioactive natural products such as curcumin (Malar and Devi, 2014), berberine (Jiang et al., 2011; Shan et al., 2011), or 8-hydroxyquinoline (Gomes et al., 2014) have been used as structural scaffolds for the development and search of new chemical entities with multiple properties or MTDLs for the treatment of AD. These new entities may be considered as simplified versions or lead drugs possessing great potential as real alternatives to the current unsuccessful pharmacological therapies for effectively fighting against AD and other complex diseases.

**Figure 6:**
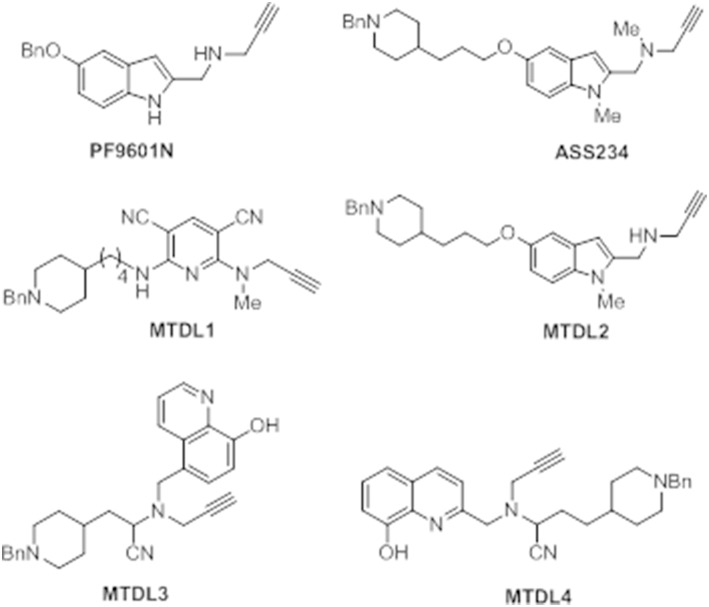
**Structure of compounds PF9601N, ASS234, and the MTDL1-4 described in this review**.

The next sections include an overview on the outcomes obtained from the biological assessment of several MTDLs molecules that have been lately reported by some of us. All the compounds described bear the *N*-benzylpiperidine group present in donepezil and the *N*-propargylamine motif present in **PF9601N**, a potent and selective MAO B inhibitor with neuroprotective effects *in vitro* and *in vivo* using different experimental models of neurodegenerative diseases (Cutillas et al., 2002; Pérez and Unzeta, 2003; Pérez et al., 2003; Battaglia et al., 2006; Sanz et al., 2009). Both scaffolds were linked by different heterocyclic ring systems, such as pyridine, indole or 8-hydroxyquinoline, allowing the facile synthesis of different MTDL molecules as promising drugs to be used in AD therapy (Figure 6). The inhibitory profile of these new MTDL molecules inhibiting ChE and MAO, their antioxidant, anti-β-aggregating, anti-inflammatory, and anti-apoptotic behavior together with their metal-chelating properties were determined and results from comparative studies were assessed. Taken together, the outcomes reported with these derivatives reveal a potential improvement of the current pharmacological therapy of AD.

### ASS234

**Figure 7:**
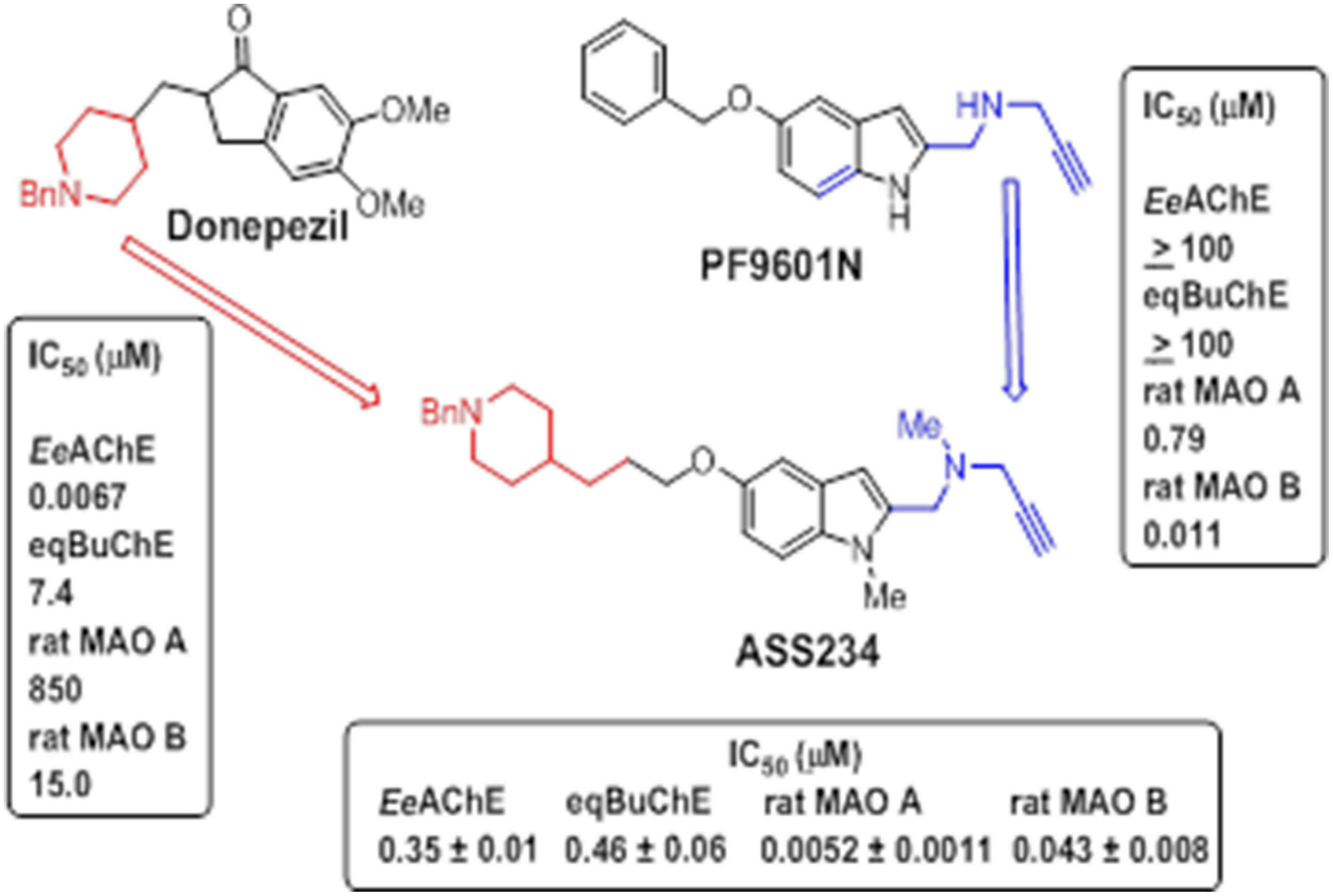
**Design strategy of ASS234**. IC_50_ values (in μM) of donepezil, **PF9601N** and **ASS234** inhibiting both ChEs and MAO activities are shown Bolea et al. (2011).

**ASS234** {*N*-[5-(3-(1-benzylpiperidin-4-yl)propoxy)-1-methyl-1*H*-indol-2-ylmethyl]-*N*-methylprop-2-yn-1-amine} (Figure 7) was first identified by some of us as a suitable candidate for use in AD as a result of extensive screening of several series of novel derivatives. This multipotent molecule was specifically developed to combine the anti-cholinergic activity of donepezil, as an AChE inhibitor widely used in AD therapy, with a propargylamine moiety derived from selective MAO B inhibitor, **PF9601N** (Figure 7) with neuroprotective properties (Bolea et al., 2011).

**ASS234** was revealed as a potent inhibitor of both MAO A and MAO B, with an IC_50_ values of 5.2 ± 1.1 nM and 43.1 ± 7.9 nM, respectively. It also inhibited both ChEs exhibiting IC_50_ values of 0.35 ± 0.001 and 0.46 ± 0.06 μM toward AChE and BuChE, respectively. In comparison with donepezil and **PF9601N**, analyzed under the same experimental conditions, donepezil was ineffective at inhibiting MAO activities whereas **PF9601N** potently and selectively inhibited MAO B isoform but displayed no interaction with both ChEs (Bolea et al., 2011). These data indicate that **ASS234** combines the desirable properties of donepezil and **PF9601N** being capable of simultaneously enhancing both cholinergic and monoaminergic transmission as well as possessing neuroprotective effects (Bolea et al., 2013).

Kinetic studies showed that **ASS234** is a-reversible inhibitor of both ChEs, with micromolar affinity, and a highly potent irreversible MAO A inhibitor with similar behavior to clorgyline (Esteban et al., 2014). The parent propargylamine, **PF9601N**, behaves similarly to *l*-deprenyl as an effective selective MAO B inhibitor. Both **PF9601N** and **ASS234** display as mechanism-based MAO inhibitors. The selectivity of **ASS234** as a MAO A inhibitor, indicated by the IC_50_ values, for both purified and membrane-bound protein samples (Pérez et al., 1999; Bolea et al., 2011), might be attributed to the shared propargylamine structure, which is larger and more hydrophobic in **ASS234**. The initial reversible binding parameter (K_*i*_ value of 0.2 μM) indicated that **ASS234** has slightly lower affinity for MAO A than clorgyline (K_*i*_ value of 0.04 μM), and this was also reflected in higher rate constant (*k*_1_) for the irreversible reaction.

UV-VIS spectral analyses showed an irreversible modification of the MAO flavin group by **ASS234** similar to that found with other propargyl MAO inhibitors. The crystal structure of human MAO B in complex with **ASS234** revealed the formation of a covalent adduct with the N5 atom of the flavin cofactor. The *N*-benzylpiperidine moiety of the compound was not able to fully bind to the intact molecule to the MAO B active site, which ruled out the possibility that the inhibitor may have undergone degradation. Thus, these outcomes demonstrated the efficacy of **ASS234** as inhibitor of both the “classic” AD-targeted ChE enzymes and the MAOs (Esteban et al., 2014).

Following the search for additional activities with multi-target **ASS234**, the potential modulation of the monoaminergic transmission and metabolism by this molecule were also investigated both *in vitro* and *in vivo* (Van Schoors et al., unpublished results). Since some of the behavioral alterations occurring in AD such as depression are possibly caused by monoaminergic dysfunction, the therapeutic use of antidepressant drugs based on the selective inhibition of MAO A, an enzyme possessing an indispensable role in the metabolic regulation of neurotransmitters 5-HT, NA, DA, and/or on the blockage of the corresponding reuptake systems at the pre-synaptic nerve endings, may also be considered.

In this context, a significant increase in the levels of 5-HT associated with a reduction in the levels of its metabolite 5-HIAA was observed as a result of irreversible inhibition of MAO A in SH-SY5Y cells treated with **ASS234**. Comparatively, similar though less pronounced effects were found with highly-selective MAO A inhibitor clorgyline under the same experimental conditions, thus revealing an active effect of this multi-target drug to in enhancing 5-HT levels. In contrast, DA levels were significantly decreased in PC12 cells that had been treated with **ASS234** for 24 h. This finding may be due to a reduction of both activity and expression of the enzymes responsible for DA synthesis, tyrosine hydroxylase and aromatic L-amino acid decarboxylase as a response to initial rapid elevation of DA levels following the blockage of MAO activity. Both MAO A inhibitors **ASS234** and clorgyline were able to modulate the levels of DA metabolites DOPAC and HVA, which were decreased after incubations. *In vivo* microdialysis studies of the effects upon administration of **ASS234** in freely-moving rats also revealed alterations in the extracellular monoamines levels in both hippocampus and prefrontal cortex, two brain areas that are highly-impaired in AD. Interestingly, the effect of **ASS234** differed from brain areas and dosage. In hippocampus, levels of 5-HT and NA were increased whereas those of DA and NA markedly augmented in prefrontal cortex.

These differences might be attributed to the presence of metabolizing enzymes in both brain areas as well as to the complexity of the monoaminergic neurochemical interactions existing in the brain. In addition, **ASS234**, as a multi-target compound, might be able to modulate other neurobiological systems and therefore producing a complex pharmacological outcome. The results from the *in vivo* studies revealed a time-dependent effect on extracellular levels of monoaminergic neurotransmitters after administration of a single, subcutaneous, dose of **ASS234**. This results could be used in further studies to assess both the pharmacokinetics and bioavailability of the compound in reaching its therapeutic target. Many factors, including route of administration and the existence of active metabolites may contribute to the observed delay in the *in vivo* effects observed with **ASS234**.

In AD, levels of DA and NA are diminished in cortex and hippocampus while those of 5-HT are decreased in hippocampus (Reinikainen et al., 1988), producing some distinctive behavioral symptoms including depressive-like disorder, psychosis, or memory impairment related to alterations in serotonergic, catecholaminergic, and cholinergic neurotransmissions (Vermeiren et al., 2015). Therefore, the outcomes observed *in vivo* upon the administration of **ASS234** in addition with those previously found, confirm the potential value of this compound to be used as a modulator of the monoaminergic transmission in AD, both in terms of therapy and for further elucidation of the mechanisms involved.

The therapeutic potential of **ASS234** has been also evaluated following its administration to a rat model of vascular dementia (Stasiak et al., 2014). These experiments involved the permanent bilateral occlusion of the common carotid arteries (BCCAO) with experimental vascular dementia. In this rat model, the administration of **ASS234** for five consecutive days resulted in a potent and selective inhibition of brain MAO A activity as well as a concurrent increase in the concentrations of serotonin and catecholamines dopamine and noradrenaline. BCCAO resulted in impaired time parameters and memory functions as measured by hole-board memory tests in rats. Treatment of BCCAO rats with **ASS234** showed less negative effects on cognitive parameters and exerted a significant positive effect on working memory.

Aggregation and deposition of Aβ is a key pathological hallmark of AD and there is increasing evidence suggesting that the neurotoxicity of this peptide is related to the formation of toxic oligomeric aggregates. In this regard, **ASS234** has demonstrated to possess anti-Aβ_1−42_ aggregating capacity and also an ability to reduce the presence of oligomeric forms of β-amyloid, as the most toxic species in AD (Bolea et al., 2013). Moreover, **ASS234** was able to inhibit AChE-catalyzed Aβ aggregation by binding to the PAS of the enzyme. **ASS234** also showed a protective effects on the Aβ_1−42_-mediated cytotoxicity induced in human neuroblastoma cells by preventing the activation of the intrinsic mitochondrial pathway of apoptosis. Moreover, **ASS234** displayed significant antioxidant properties, arising from the increase in the expression of catalase and SOD-1 in human neuroblastoma SH-SY5Y cells. This compound also has the capacity to capture free radicals *in vitro*, as measured by the well-established ORAC-FL (Oxygen radical Absorbance Capacity) fluorescent method. It is noteworthy that in a recent *in vivo* approach using transgenic APP/PS1AE9 mice given **ASS234**, both Aβ plaques in cortex and activated glia were found decreased (Serrano et al., 2016).

**Figure 8:**
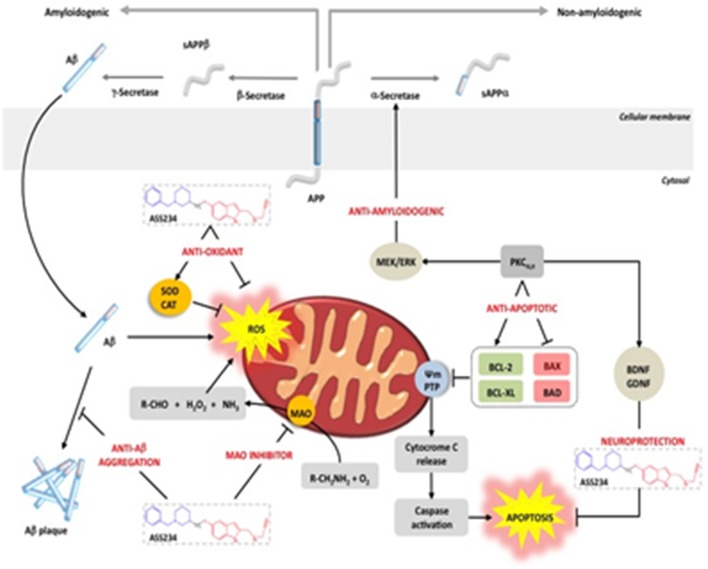
**Schematic representation of ASS234 targets involved in AD pathogenesis. ASS234** forms a N5 flavin adduct (like clorgyline) with MAO A and it is able to block AChE-induced Aβ aggregation. **ASS234** shows antioxidant and anti-apoptotic properties and it is able to induce neuroprotection through the Wnt pathway. **ASS234** also shows less toxicity than donepezil in HepG2 cells (With permission of Bolea et al., 2013b).

These findings, summarized in Figure 8, allow us to conclude that **ASS234** is able to interact with multiple targets relevant to AD. They also indicate that this is an interesting MTDL molecule that should be considered for therapeutic development against AD.

### Derivatives MTDL-1 and MTDL-2 (*donepezil-pyridyl and indolyl-propargyl hybrids*) as dual ChE/MAO inhibitors

**Figure 9:**
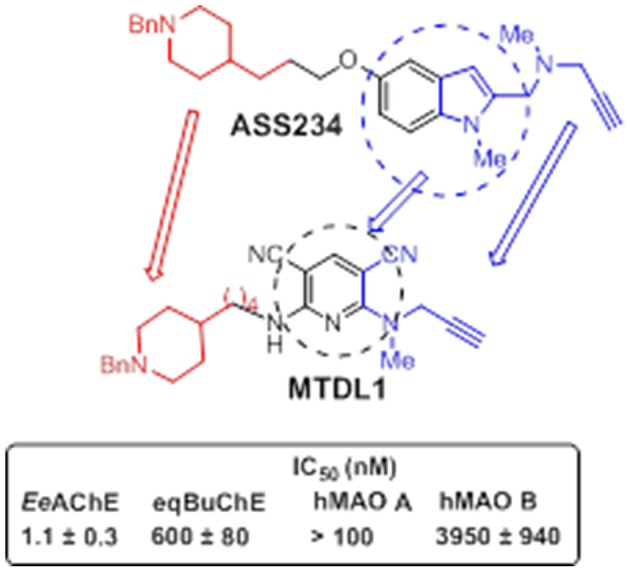
**Designed structure of hybrid MTDL1 and IC_**50**_ values for the inhibition of ChE and MAO enzymes (Bautista-Aguilera et al., 2014a)**.

**Figure 10:**
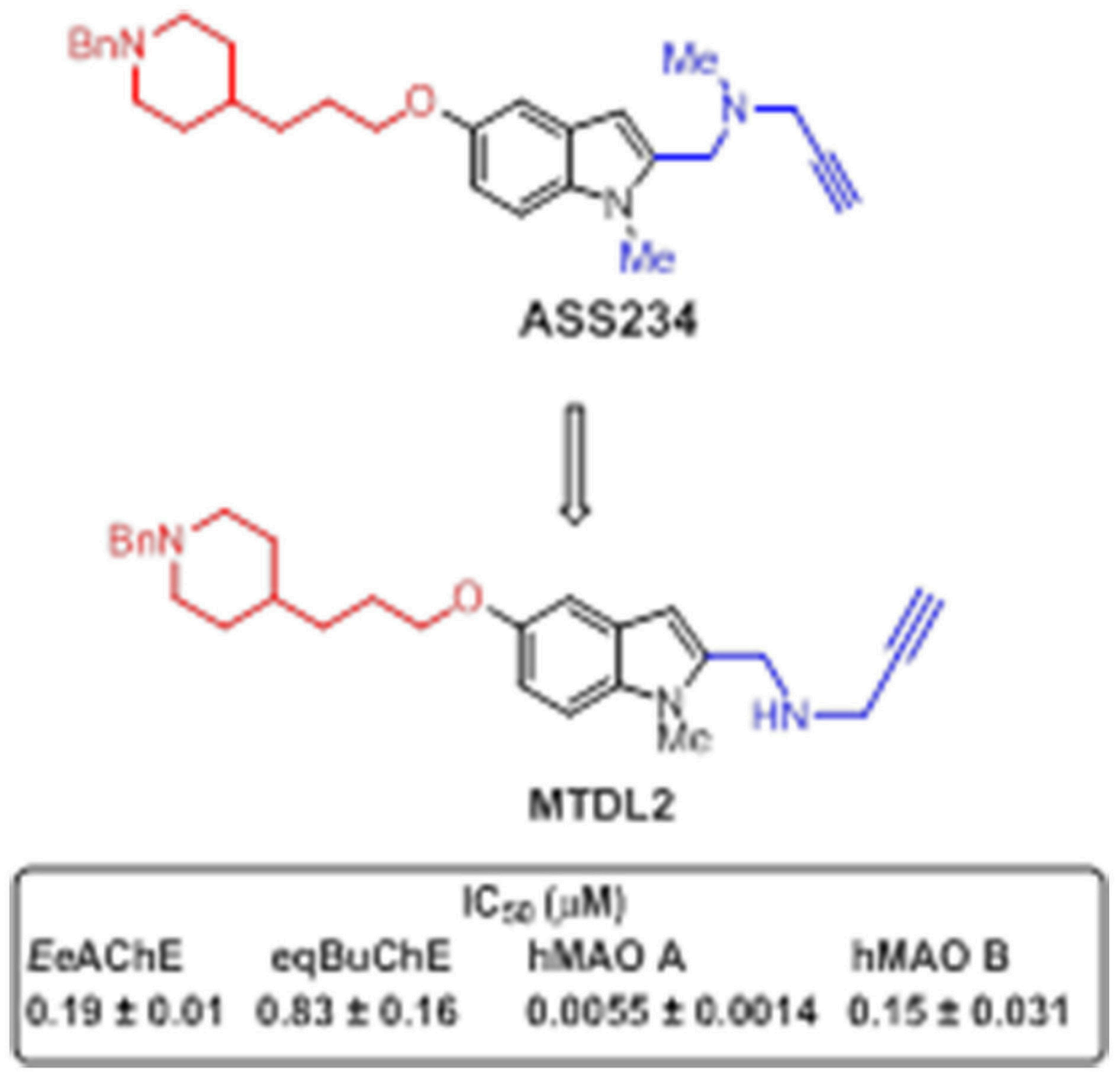
**Structure of hybrid MTDL2 and IC_**50**_ values for the inhibition of ChEs and MAO enzymes (Bautista-Aguilera et al., 2014b)**.

With the aim of searching for improved MTDLs, two series of novel structurally-derived compounds, derived from **ASS234** as multipotent donepezil-indolyl and donepezil-pyridyl hybrids were designed and pharmacologically evaluated for their potential use in AD. The synthetic strategy that was used to design these novel donepezil-pyridyl hybrids is outlined in Figures 9, 10.

Among all the compounds tested, the donepezil-pyridyl hybrid **MTDL-1** (Figure 9) was identified as a potent AChE inhibitor (IC_50_ = 1.1 nM) and a moderate BuChE inhibitor (IC_50_ = 0.6 μM) with total selectivity toward human MAO B (Bautista-Aguilera et al., 2014a). Interestingly, the design and development of non-selective ChE inhibitors for use in AD appears of valuable pharmacological concern, as the hydrolysis of neurotransmitter ACh may largely occur via BuChE catalysis in aged AD brains, the activity levels of which have been found elevated on late-stages of the disease as it is also the case with MAO B. Since BuChE is also found in glial cells that are recruited and activated around the plaques and tangles, the inhibition of this enzyme might provide additional benefits at reducing neuroinflammation. Suitable drug-likeness profile and ADMET properties of these two novel MTDLs were also confirmed by 3D-QSAR studies.

The donepezil-indolyl hybrid molecule **MTDL-2** (Figure 10), exhibited an interesting profile as potent MAO A inhibitor (IC_50_ = 5.5 nM) that was moderately able to inhibit MAO B (IC_50_ = 150 nM), AChE (IC_50_ = 190 nM), and BuChE (IC_50_ = 830 nM) (Bautista-Aguilera et al., 2014b). Moreover, this compound appeared to be a mixed-type AChE inhibitor able to span both the CAS and PAS of this enzyme, as found by molecular modeling studies. Interestingly, docking simulations revealed that the selective inhibition of MAO isoforms by propargyl-containing compounds accounts for the orientation on their propargylamine and phenyl moieties in MAO, which energetically affects the interaction with the active site.

### Derivatives MTDL-3 and MTDL-4 (*donepezil+propargylamine+8-hydroxyquinoline hybrids*) as dual ChE/MAO inhibitors with antioxidant activity, metal-chelating properties, and other pharmacological targets

**Figure 11:**
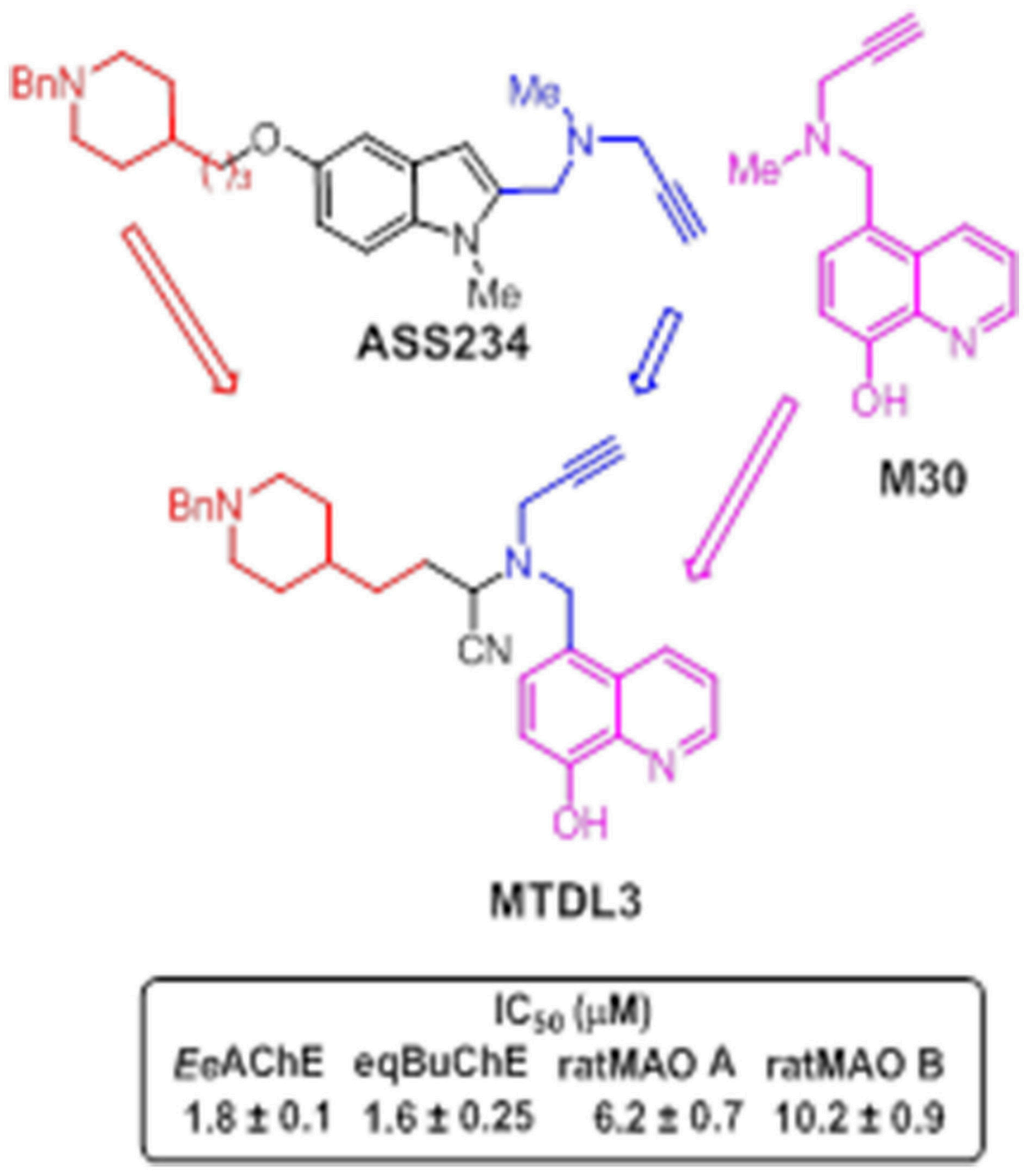
**Structure of hybrid MTDL3 and IC_**50**_ values for the inhibition of ChEs and MAO enzymes (Wang et al., 2014)**.

**Figure 12:**
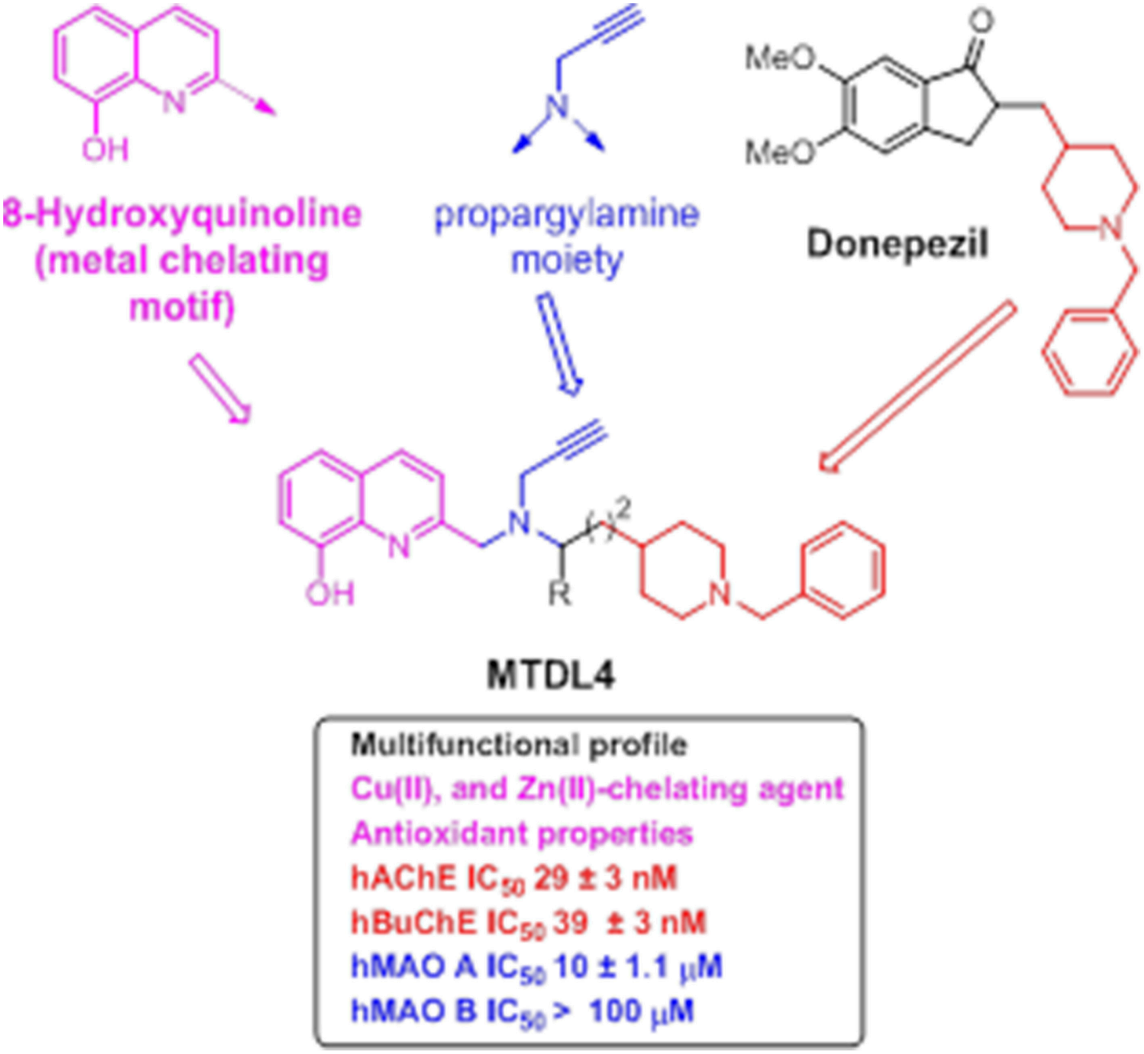
**Structure of hybrid MTDL-4 and IC_**50**_ values for the inhibition of ChEs and MAO enzymes (Wu et al., 2015)**.

Newly-designed **MTDL-3** and **MTDL-4** derivatives containing the *N*-benzylpiperidine moiety from donepezil and a metal-chelating 8-hydroxyquinoline group (Figures 11, 12), were linked to a central *N*-propargylamine core and pharmacologically evaluated (Wang et al., 2014; Wu et al., 2015). This molecule contains a moiety also present in the compound M30, a brain-permeable and iron-chelating compound with antioxidant activity displaying neuroprotective activity in animal models (Salkovic-Petrisic et al., 2015). M30 modulates HIF-α-related glycolytic genes in the frontal cortex of APP/PS1 mice used as AD model (Mechlovich et al., 2014). These studies also showed M30 to have beneficial effects on several major hallmarks of AD (Kupershmidt et al., 2012), indicating the potential value of incorporating this moiety into **MTDL-3** (Figure 11).

**MTDL-3** was revealed as an irreversible MAO inhibitor and mixed-type ChE inhibitor, in low micromolar range, able to strongly complex Cu (II), Zn (II), and Fe (III) (Wang et al., 2014). In addition, the cyano group, which was only present in some derivatives, was found to enhance the binding to AChE, BuChE, and MAO A (Wang et al., 2014). Although α-aminonitriles have seldom been investigated as ChE inhibitors, some previous studies described nitriles as MAO inhibitors, and reported that cyanide potentiates irreversible MAO inhibition (Ramadan et al., 2007). Although cyanide is known to be a poor reversible inhibitor of the oxidation of benzylamine by MAO (Houslay and Tipton, 1973), previous studies have indicated that the inhibitory activity of several compounds against MAO is potentiated by cyanide (Davison, 1957; Ramadan and Tipton, 1998; Ramadan et al., 1999, 2007; Juárez-Jiménez et al., 2014). An earlier study by Davison (1957) on the inhibition of mitochondrial MAO by the irreversible inhibitor iproniazid revealed that, although at low concentrations, this compound alone had little effect on enzyme activity, while the inhibitory activity was significantly enhanced in the presence of cyanide ions. A similar potentiating effect was also reported for phenelzine and pheniprazine (Ramadan et al., 1999). Such studies demonstrated that, in case of pheniprazine, cyanide potentiates the inhibition by increasing the apparent binding affinity to MAO without producing significant changes in the rate of the reaction that yields the irreversibly inhibited species (Ramadan et al., 2007). This finding suggests that the potentiating effect of cyanide can be ascribed to an activation mechanism whereby cyanide assists the binding of the inhibitor to the enzyme.

Theoretical ADMET analyses, showed **MTDL-3** to exhibit good drug-likeness properties and brain penetration capacity for CNS activity. Moreover, less toxicity than donepezil in HepG2 cells was also revealed with these derivatives. These findings are important, of noteworthy relevance since the simultaneous administration of multiple therapeutic agents (polypharmacology) may lead to potentially lethal side effects triggered by drug-to-drug interactions. Therefore, such undesirable outcomes might be significantly reduced by the use of multi-target derivatives such as **MTDL-3**. Furthermore, these molecules also displayed good performance in terms of translational science since scopolamine-induced memory deficits were partially restored by **MTDL-3**, confirming its effective pharmacokinetics *in vivo*.

Another related molecule, **MTDL-4** (Figure 12), showed similar behavior as dual ChE/MAO inhibitor (Wu et al., 2015), which was identified from an initial pharmacological screening as an appropriate lead compound for further investigation. Subsequent investigations revealed further potentially valuable properties of this molecule.

## Concluding remarks

Overall, the findings presented in this review robustly reinforce the suitability of MTDLs as an appropriate pharmacological approach to target disease progression in AD therapy. Amongst all the compounds tested, multi-target **MTDL-3** particularly emerged as a ligand possessing a number of remarkable potential benefits for use in this neurological disorder including well-balanced dual AChE/MAO inhibition, strong metal-chelating activity, neuroprotective and anti-apoptotic properties, potent antioxidant capacities and anti-inflammatory action. Furthermore, **MTDL-3** has effects as enhancer of cognitive functions. This prompts us to propose this molecule as the first racemic α-aminonitrile identified so far as a multifunctional chelator for biometals able to interact in two key enzymatic systems implicated in AD. We consider it as an important new lead compound that merits further investigation for the potential treatment of this disease.

## Author contributions

MU, GE, JM, wrote the manuscript. RR, KT, and WF, corrected and revised the manuscript.

### Conflict of interest statement

The authors declare that the research was conducted in the absence of any commercial or financial relationships that could be construed as a potential conflict of interest.

## Abbreviations

AD: Alzheimer's disease
A*β*: amyloid beta
NFT: neurofibrillary tangles
AChE: acetylcholinesterase
BuChE: butyrylcholinesterase
MAO: monoamine oxidase
APP: amyloid precursor protein
ROS: reactive oxygen species
MTDL: multi-target-directed ligand.
